# Intermediate- vs. Standard-Dose Prophylactic Anticoagulation in Patients With COVID-19 Admitted in Medical Ward: A Propensity Score-Matched Cohort Study

**DOI:** 10.3389/fmed.2021.747527

**Published:** 2021-10-15

**Authors:** David M. Smadja, Guillaume Bonnet, Nicolas Gendron, Orianne Weizman, Lina Khider, Antonin Trimaille, Tristan Mirault, Charles Fauvel, Jean-Luc Diehl, Delphine Mika, Aurelien Philippe, Théo Pezel, Guillaume Goudot, Willy Sutter, Benjamin Planquette, Victor Waldmann, Olivier Sanchez, Ariel Cohen, Richard Chocron

**Affiliations:** ^1^Innovative Therapies in Haemostasis, INSERM, Université de Paris, Paris, France; ^2^Department of Hematology and Biosurgical Research Lab (Carpentier Foundation), AP-HP, Georges Pompidou European Hospital, Paris, France; ^3^F-CRIN INNOVTE, Saint-Étienne, France; ^4^Paris Cardiovascular Research Center (PARCC), INSERM, Université de Paris, Paris, France; ^5^Center Hospitalier Universitaire de Bordeaux, Hôpital Cardiologique Haut-Lévêque, Unité Médico-Chirurgical de Valvulopathies et Cardiomyopathies, Pessac, France; ^6^Institut Lorrain du Coeur et des Vaisseaux, CHU de Nancy, Vandoeuvre les Nancy, France; ^7^Department of Vascular Medicine, AP-HP, Georges Pompidou European Hospital, Paris, France; ^8^Nouvel Hôpital Civil, Center Hospitalier Régional Universitaire de Strasbourg, Strasbourg, France; ^9^Rouen University Hospital, FHU REMOD-VHF, Rouen, France; ^10^Medical Intensive Care Department and Biosurgical Research Lab (Carpentier Foundation), AP-HP, Georges Pompidou European Hospital, Paris, France; ^11^INSERM, Université Paris-Saclay, Chatenay-Malabry, France; ^12^Department of Cardiology, Lariboisière Hospital, AP-HP, University of Paris, Paris, France; ^13^Department of Vascular Surgery, AP-HP, Georges Pompidou European Hospital, Paris, France; ^14^Department of Pneumology and Intensive Care and Biosurgical Research Lab (Carpentier Foundation), AP-HP, Georges Pompidou European Hospital, Paris, France; ^15^Department of Cardiology, AP-HP, Georges Pompidou European Hospital, Paris, France; ^16^Department of Cardiology, AP-HP, Saint Antoine Hospital, Paris, France; ^17^Department of Emergency, AP-HP, Georges Pompidou European Hospital, Paris, France

**Keywords:** SARS-CoV-2, anticoagulation, intermediate dose, prophylactic treatment, mortality, COVID-19, LMWH

## Abstract

**Background:** Microthrombosis and large-vessel thrombosis are the main triggers of COVID-19 worsening. The optimal anticoagulant regimen in COVID-19 patients hospitalized in medical wards remains unknown.

**Objectives:** To evaluate the effects of intermediate-dose vs. standard-dose prophylactic anticoagulation (AC) among patients with COVID-19 hospitalized in medical wards.

**Methods and results:** We used a large French multicentric retrospective study enrolling 2,878 COVID-19 patients hospitalized in medical wards. After exclusion of patients who had an AC treatment before hospitalization, we generated a propensity-score-matched cohort of patients who were treated with intermediate-dose or standard-dose prophylactic AC between February 26 and April 20, 2020 (intermediate-dose, *n* = 261; standard-dose prophylactic anticoagulation, *n* = 763). The primary outcome of the study was in-hospital mortality; this occurred in 23 of 261 (8.8%) patients in the intermediate-dose group and 74 of 783 (9.4%) patients in the standard-dose prophylactic AC group (*p* = 0.85); while time to death was also the same in both the treatment groups (11.5 and 11.6 days, respectively, *p* = 0.17). We did not observe any difference regarding venous and arterial thrombotic events between the intermediate dose and standard dose, respectively (venous thrombotic events: 2.3 vs. 2.4%, p=0.99; arterial thrombotic events: 2.7 vs. 1.2%, *p* = 0.25). The 30-day Kaplan–Meier curves for in-hospital mortality demonstrate no statistically significant difference in in-hospital mortality (HR: 0.99 (0.63–1.60); *p* = 0.99). Moreover, we found that no particular subgroup was associated with a significant reduction in in-hospital mortality.

**Conclusion:** Among COVID-19 patients hospitalized in medical wards, intermediate-dose prophylactic AC compared with standard-dose prophylactic AC did not result in a significant difference in in-hospital mortality.

## Introduction

More than respiratory disease, coronavirus disease 2019 (COVID-19) caused by severe acute respiratory syndrome coronavirus 2 (SARS-CoV-2) is a systemic acquired vascular disease associated with inflammation (1), endothelial injury (2), and high thrombosis prevalence, in particular pulmonary embolism (PE) (3–8). *In situ* microthrombosis has emerged as a key feature of COVID-19, in contrast to an embolus, commonly observed in deep vein thrombosis. Microthrombosis in COVID-19 has been observed in all postmortem lung examinations and could be explained, at least in part, by the large von Willebrand factor (VWF) released following endothelial activation (9). A massive release of plasma VWF is associated with an increase of the high-molecular-weight multimers, and a slight decrease in ADAMTS13 (a disintegrin and metalloprotease with thrombospondin type I repeats-13) levels or function, that is likely drive to the generation of microthrombosis in COVID-19 (10). More so than real thrombotic complication i.e., macrothrombosis, microthrombosis is probably an important trigger of pathophysiology and, in particular, hypoxemia. Thus, prophylactic anticoagulation (AC) is likely to be one of the best treatments to avoid the worsening of the disease. Empirically, higher prophylactic dosing of unfractionated heparin or low molecular weight heparin (LMWH) has become relatively common to limit the formation of microthrombi according to the second version of the International Society on Thrombosis and Haemostasis (11) and the French guidelines (12). Therapeutic AC should be administered only if a thrombotic event or PE is occurring. In a recent multicenter randomized trial (13), intermediate-dose in contrast to standard-dose prophylactic AC did not improve outcomes, including mortality in patients admitted in the intensive care units (ICU). The aim of our study was to investigate the effect on in-hospital mortality of intermediate- vs. standard-dose prophylactic AC in patients with COVID-19 admitted in medical wards using a propensity score-matched cohort study.

## Methods

Data will be made available from the authors on reasonable request.

### Study Settings and Population

From February 26 to April 20, 2020, consecutive patients with a diagnosis of SARS-CoV-2 infection and initially hospitalized in medical wards were included (none of the patients were directly admitted to ICU). Patients were aged over 18 years and were included in a retrospective, multicenter (24 centers), observational cohort study, which was named the critical COVID-19 France (CCF) study, which initiated by the French Society of Cardiology. Following World Health Organization (WHO) criteria, SARS-CoV-2 infection was determined by a positive result from a real-time reverse transcriptase-polymerase chain reaction (RT-PCR) test of nasal or pharyngeal swabs, or lower respiratory tract aspirates (confirmed case), or by typical imaging characteristics on chest computed tomography (CT) when laboratory testing was inconclusive. The CCF study was declared to and authorized by the French data protection committee (authorization no. 2207326v0), and conducted in accordance with the ethical standards established in the Declaration of Helsinki and its later amendments (NCT04344327) (7, 14, 15).

### Data Collection and AC Regimen

All data were retrospectively collected by local investigators in an electronic case-report form *via* the REDCap® software (Research Electronic Data Capture, Vanderbilt University, USA) hosted by a secured server from the French Institute of Health and Medical Research at the Paris Cardiovascular Research Center. Baseline information of the patient included demographic characteristics, coexisting medical conditions, cardiovascular comorbidities, and chronic medications. Clinical parameters and biological findings were recorded at admission. On the chest CT scan, the degree of pulmonary lesions with ground-glass opacities and areas of consolidation were categorized as low/moderate (<50% involvement) or severe (≥50% involvement). Data on pharmacological therapies, mode of respiratory support, complications, and final vital status were also gathered throughout the hospitalization. Patients who had an AC treatment (whatever the regimen) before hospitalization were excluded from the analysis.

The AC regimens analyzed during hospitalization were categorized into two groups: standard-dose prophylactic (once daily LMWH; subcutaneous heparin injection or intravenous heparin infusions) or intermediate-dose prophylactic (twice daily LMWH, subcutaneous heparin injection or intravenous heparin infusions). The exact dosing of AC was not reported in the database and was reported according to the treating clinician. During the study period, the dose of the AC regimen was based on the International Society on Thrombosis and Haemostasis (11) and the French guidelines (12). The time from hospitalization (Day 0) to death was used as an outcome. Symptomatic venous and ischemic thrombotic events and outcomes (in-hospital death) were assessed using electronic medical records.

### Statistical Analysis

To address confounding and other sources of bias arising from the use of observational data, we estimated a propensity-matched analysis for the likelihood of treatment with intermediate-dose prophylactic AC. We estimated the propensity score by running a logistic regression model where the outcome variable is a binary variable indicating treatment status (intermediate-dose or standard-dose prophylactic anticoagulation), including as covariates the following: age, sex, body mass index, high blood pressure, diabetes mellitus, dyslipidemia, current smoking status, and history of cancer. Then a 1:3 match was performed using Greedy matching techniques. All analyses were performed on matched populations. We used standardized mean difference (SMD), which is the most commonly used statistic to examine the balance of covariate distribution between the two groups (intermediate-dose or standard-dose prophylactic anticoagulation). Continuous data were expressed as median (interquartile range, IQR) and categorical data as *n* (%). Patients were compared according to the AC regimen during hospitalization (intermediate-dose or standard-dose prophylactic AC). In the multivariable analysis, we used conditional logistic regression to assess the association between the AC regimen and outcomes. The interactions between the AC regimen and specific subgroups were assessed *via* the Cochran–Mantel–Haenszel *X*^2^ test. For the survival analysis, the start of the study was triggered by the diagnosis of SARS-CoV-2 infection and hospitalization in a medical ward. The end of the study was defined either by the death of the patient during the hospitalization or by discharge alive from the hospital. We used the Cox proportional hazard (PH) model to investigate the relationships between AC regimen and outcomes. Missing data were handled using multiple random forest imputations using chained equations (10 sets of imputations). All analyses were two-sided and a *p* < 0.05 was considered statistically significant. Statistical analysis was performed using R studio® software including R version 3.6.3 (R Development Core Team, 2019).

## Results

During the study period, 2,878 consecutive patients were hospitalized for SARS-CoV-2 infection in medical wards and were included, as previously described (7, 14, 15). We excluded 382 patients from this analysis who had an AC treatment (whatever the regimen) before hospitalization. Among the study population, 261 (25%) patients started an intermediate-dose of prophylactic AC during hospitalization. The propensity matching yielded 783 (75%) patients who received standard-dose and 261 (25%) patients who received intermediate-dose prophylactic AC during hospitalization, with balanced confounders between the groups.

**Figure 1:**
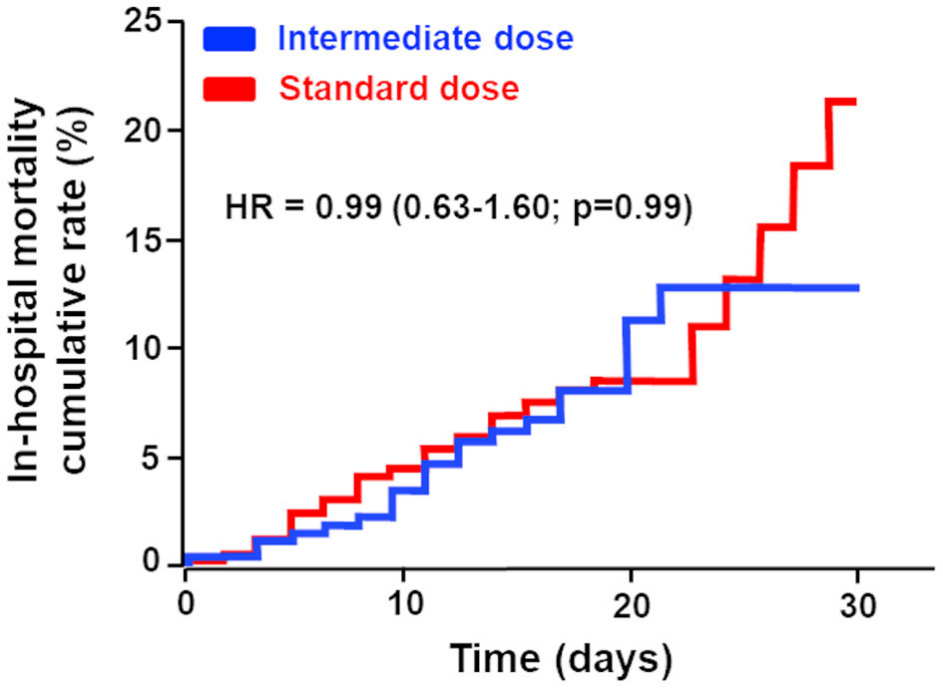
Cumulative in-hospital mortality rate of patients with COVID-19 admitted to medical wards treated either by intermediate-dose vs. standard-dose prophylactic. The primary outcome was in-hospital mortality. The median (interquartile range) follow-up time was 12 [8.0–19.0] days in the intermediate-dose group and 8 [5.0–12.6] days in the standard-dose prophylactic anticoagulation group.

As shown in Table 1, the study population had a median age of 63.7 (IQR 51.9–74.2) years, 391 (37.5%) patients were women, and the median body mass index was 28.6 (IQR 25.4–32.2). As expected according to international and French guidelines about the use of intermediate doses of prophylactic AC, fewer patients received the standard dose, and these had less lung extensive damage on CT scan, had decreased C-reactive protein (CRP) at admission, and received less high-flow nasal cannula or endotracheal intubation during follow-up, in contrast with patients who received an intermediate dose of prophylactic AC. In-hospital mortality occurred in 23 of 261 (8.8%) patients in the intermediate-dose group and 74 of 783 (9.4%) patients in the standard-dose prophylactic AC group (*p* = 0.85); while time to death was also the same in both treatment groups (11.3 [8.1–13.5] and 11.6 [10.2–12.5] days, respectively, *p* = 0.17). Moreover, the 30-day Kaplan–Meier curves for in-hospital mortality, shown in Figure 1, demonstrated no statistically significant differences in in-hospital mortality (hazard ratio: 0.99; confidence interval 0.63–1.60; *p* = 0.99). Furthermore, we did not observe any difference regarding venous and arterial thrombotic events between intermediate dose and standard dose (respectively, venous thrombotic events: 2.3 vs. 2.4%, *p* = 0.99; arterial thrombotic events: 2.7 vs. 1.2%, *p*=0.25). We finally performed a subgroup analysis and found that no particular subgroups were identified in which the use of any AC regimen was associated with a significant reduction in the primary outcome (Table 2). We did not observe any interaction between potential confounders and the AC regimen (Table 2).

**Table 1:** Baseline characteristics of the matched population between intermediate- vs. standard-dose prophylactic anticoagulation among patients with COVID-19 admitted in medical wards.

	**Whole population (*n* = 1,044)**	**Prophylactic anticoagulation**			
		**Intermediate dose**	**Standard dose**	***p*-value**	**SMD**
		**(*n* =261)**	**(*n* = 783)**		
Age, *median [IQR]*	63.7 [52.0–74.2]	63.0 [52.7–73.7]	63.9 [51.2–74.5]	0.78	0.016
**Gender**, ***n*** **(%)**					
Women	391 (37.5)	100 (38.3)	300 (38.3)	1.00	0.024
Men	653 (62.5)	161 (61.7)	483 (61.7)		
Body Mass Index, *median [IQR]*	28.6 [25.4–32.2]	28.6 [25.7–32.3]	28.7 [25.5–32.2]	0.77	0.043
Time from illness onset to hospitalization, *median [IQR]*	7.0 [4.0, 10.0]	7.0 [4.0, 9.0]	7.00 [4.0, 10.0]	0.29	0.076
Lung extensive damage on CT scan >50%	179 (17.1)	85 (32.6)	104 (13.3)	<0.001	0.033
**Coexisting conditions**, ***n*** **(%)**					
Hypertension	474 (45.4)	120 (46.0)	363 (46.4)	0.97	0.015
Diabetes	291 (27.9)	77 (29.5)	224 (28.6)	0.84	0.037
Hyperlipidemia	275 (26.3)	69 (26.4)	197 (25.2)	0.74	0.003
Peripheral arterial disease	42 (4.0)	11 (4.2)	25 (3.2)	0.56	0.013
Ischemic stroke	57 (5.5)	12 (4.6)	36 (4.6)	1.00	0.052
Kidney failure	113 (10.8)	35 (13.4)	75 (9.6)	0.10	0.107
Cancer					
No cancer	918 (87.9)	228 (87.4)	678 (86.6)	0.94	0.026
Active cancer	52 (5.0)	14 (5.4)	43 (5.5)		
Cancer remission	74 (7.1)	19 (7.3)	62 (7.9)		
Current smoker	167 (16.0)	40 (15.3)	119 (15.2)	1.00	0.025
Thromboembolic disease					
None	998 (95.6)	248 (95.0)	746 (95.3)	0.36	0.087
Arterial thrombosis	9 (0.9)	4 (1.5)	5 (0.6)		
VTE	41 (4.0)	9 (3.4)	32 (4.1)		
Atrial fibrillation	39 (3.7)	11 (4.2)	21 (2.7)	0.30	0.007
					
**Medication history**, ***n*** **(%)**					
Antiplatelet therapy	244 (23.4)	62 (23.8)	172 (22.0)	0.61	0.012
Angiotensin-converting enzyme (ACE) inhibitors	176 (16.9)	46 (17.6)	129 (16.5)	0.74	0.027
Renin-angiotensin-aldosterone system inhibitors	164 (15.7)	41 (15.7)	128 (16.3)	0.88	<0.001
**Laboratory values at baseline**, ***median [IQR]***					
Hemoglobin level, g/L	1,350 [123.8–146.2]	134.0 [120.0–145.0]	135.0 [123.0–147.0]	0.17	0.141
Platelet count, x109/L	203 [157–266]	202 [157–263]	199 [155–265]	0.71	0.034
White blood cell count, x109/L	6.40 [4.8–8.7]	6.8 [4.9–9.0]	6.24 [4.7–8.2]	0.09	0.080
Glomerular filtration rate by Cockcroft	99.6 [67.1–134.2]	98.0 [66.4–131.0]	98.5 [67.1–132.2]	0.57	0.062
C-reactive protein, mg/dL	75.0 [38.0–134.1]	95.0 [50.0–152.0]	70.8 [32.7–127.0]	<0.001	0.277
**Acute respiratory support**, ***n*** **(%)**					
High flow nasal cannula	77 (7.4)	43 (16.5)	31 (4.0)	<0.001	0.405
Noninvasive positive pressure ventilation	34 (3.3)	13 (5.0)	22 (2.8)	0.14	0.120
Endotracheal intubation	157 (15.0)	82 (31.4)	82 (10.5)	<0.001	0.562
**Outcomes**, ***n (%)***					
In-hospital mortality	93 (8.9)	23 (8.8)	74 (9.5)	0.85	0.004
Time to death (median [IQR])	11.2 [9.4–12.3]	11.3 [8.1–13.5]	11.6 [10.2–12.5]	0.17	0.107
**Thrombotic events**, ***n (%)***					
VTE	25 (2.4)	6 (2.3)	19 (2.4)	0.99	0.008
PE	21 (2.1)	5 (1.9)	16 (2.0)	0.99	
DVT	9 (0.8)	3 (1.1)	6 (0.8)	0.85	
Ischemic Thrombotic Events	17 (1.6)	7 (2.7)	10 (1.2)	0.25	0.10
Myocardial infarction	9 (0.8)	4 (1.9)	5 (0.6)	0.34	
Cerebrovascular accident	5 (0.5)	3 (1.1)	2 (0.2)	0.08	
Acute limb ischemia	3 (0.2)	0 (0.0)	3 (0.4)	0.74	

*IQR, interquartile range; SMD, Standard Means Difference;. VTE, venous thromboembolic event; PE, pulmonary embolism; DVT, deep venous thrombosis.

**Table 2:** Subgroup analysis for COVID-19 in-hospital mortality: the study of the interaction between the treatment group and each of the assessed variables for in-hospital mortality.

	**Prophylactic anticoagulation**			
	**Intermediate dose**	**Standard dose**	**Unadjusted OR (95%CI, *p*-value)**	**Interaction term**
**No. of non-survivors/No. total (%)**	**23/261 (8.8)**	**74/783 (9.4)**		
Age, years				
<65	6/133 (4.5)	12/389 (3.1)	-	
≥65	17/128 (13.2)	62/394 (15.7)	4.99 (3.01–8.71, p <0.001)	2.11 (0.62–6.82, *p* =0.22)
Gender				
Women	7/100 (7)	27/300 (9.0)	-	
Men	16/161 (9.9)	47/483 (9.7)	1.17 (0.76–1.82, *p* = 0.49)	0.73 (0.24–2.05, *p* = 0.56)
Body mass index, Kg/m^2^				
<25	10/51 (19.6)	20/164 (12.2)	-	
≥25	13/210 (6.2)	54/619 (8.7)	2.54 (2.35–2.87, *p* =0.009)	2.58 (0.90–7.32, *p* = 0.08)
**Coexisting conditions**, ***n*** **(%)**				
Current smoker	3/40 (7.5)	10/119 (8.4)	0.85 (0.44–1.51, *p* = 0.60)	1.32 (0.35–6.50, *p* = 0.70)
Hypertension	18/120 (15.0)	47/363 (12.9)	2.57 (1.66–4.04, *p* <0.001)	0.70 (0.20–2.12, *p* = 0.55)
Diabetes mellitus	11/77 (14.3)	29/224 (12.9)	1.84 (1.20–2.82, *p* =0.005)	0.58 (0.21–1.62, *p* = 0.30)
Hyperlipidemia	10/69 (14.5)	21/197 (10.7)	1.42 (0.90–2.22, *p* = 0.13)	0.65 (0.24–1.83, *p* = 0.41)
Peripheral arterial disease	1/11 (9.1)	5/25 (20.0)	2.02 (0.74–4.65, *p* = 0.13)	2.49 (0.33–51.73, *p* = 0.44)
Ischemic stroke	2/12 (16.7)	7/36 (19.4)	2.38 (1.05–4.87, *p* =0.03)	1.28 (0.25–9.79, *p* = 0.78)
Kidney failure	6/35 (17.1)	23/75 (30.7)	4.56 (2.76–7.40, *p* <0.001)	1.78 (0.57–6.01, *p* = 0.33)
Atrial fibrillation	3/11 (27.2)	4/21 (19.0)	1.06 (0.31–2.72, *p* = 0.91)	1.04 (0.21–6.06, *p* = 0.97)
Cancer				
No cancer	19/228 (8.3)	57/678 (8.4)	-	
Active cancer	1/14 (7.1)	5/43 (11.6)	2.48 (1.31–4.45, *p* = 0.003)	2.61 (0.36–53.73, *p* = 0.41)
Cancer in remission	3/19 (15.8)	12/62 (19.4)	1.28 (0.48–2.87, *p* = 0.58)	1.27 (0.30–6.69, *p* = 0.76)
Thromboembolism disease				
None	22/248 (8.8)	70/746(9.4)	-	
Arterial thrombosis	0/4 (0.0)	1/5 (20.0)	1.23 (0.07–6.79, *p* = 0.85)	0.97 (0.10–17.75, *p* = 0.98)
Venous thrombosis	1/9 (11.1)	3/32 (9.4)	1.06 (0.31–2.72, *p* = 0.91)	1.28 (0.07–7.48, *p* = 0.82)
**Acute respiratory support**, ***n*** **(%)**				
High flow nasal cannula	4/43 (9.3)	5/34 (14.7)	1.07 (0.30–3.05, *p* = 0.90)	1.69 (0.37–8.01, *p* = 0.49)
Non-invasive positive pressure ventilation	1/13 (7.7)	2/21 (9.5)	0.86 (0.05–4.67, *p* = 0.88)	1.25 (0.10–29.63, *p* = 0.86)
Endotracheal intubation	9/82 (10.9)	13/75 (17.3)	1.45 (0.58–3.47, *p* = 0.41)	1.65 (0.55–5.01, *p* = 0.37)
**Medication history**, ***n*** **(%)**				
Antiplatelet therapy	10/62 (16.1)	35/172 (20.4)	3.47 (2.25–5.33, *p* <0.001)	1.40 (0.51–3.92, *p* = 0.52)
Angiotensin-converting enzyme inhibitors	5/46 (10.9)	16/129 (12.4)	1.42 (0.83–2.34, *p* = 0.18)	1.17 (0.36–4.19, *p* = 0.80)
Renin-angiotensin-aldosterone system inhibitors	6/41 (14.6)	13/128 (10.1)	1.29 (0.74–2.16, *p* = 0.34)	0.61 (0.19–2.09, *p* = 0.41)

*95%CI, 95% confidence interval; OR, odds ratio.

## Discussion

Establishing the appropriate AC prophylactic regimen in COVID-19 is an emergency according to the high rates of thrombotic complication and relevance of microthrombosis/lung obstruction observed in respiratory functional exploration (16) or autopsy studies (17). A prophylactic dose of heparin/LMWH has been used and was proposed very early in the COVID-19 outbreak after the description of their beneficial effects on mortality in the Chinese population (18). However, as thrombotic complications persisted even after a standard dose of prophylactic AC regimen, empirically, higher prophylactic dosing of heparin/LMWH has been proposed with either classic evidence-based medicine approaches or an evaluation of safety in appropriate clinical trials. Our present study suggests the futility of an increased dose for prophylactic AC regimen in patients hospitalized for COVID-19 in medical wards, as previously described by Vaughn et al. (19). We used a propensity score-matched population to compare both AC regimens in this retrospective cohort study. However, it is obvious that our data need to be confirmed in a prospective, randomized, controlled study that will also take into consideration efficacy and safety, i.e., bleeding during hospitalization with both AC regimen strategies.

One potential explanation for the absence of efficacy of intermediate-dose AC may have been the lack of intensity to prevent micro- or macrothrombosis compared with the standard-dose prophylactic regimen. We can now affirm that this is not the case, since, in critically ill COVID-19 patients enrolled in three pivotal trials testing therapeutic-dose vs. standard prophylactic AC (ACTIV-4a, REMAP-CAP, and ATTACC), the authors reported the absence of efficacy, and an increase in the frequency of the bleeding event was demonstrated (20). Similar results have been observed in a recently published INSPIRATION prospective randomized study in critically ill COVID-19 patients after a 30-day evaluation (13). Furthermore, intermediate-dose compared with standard-dose prophylactic AC did not reduce either a composite outcome of death, treatment with extracorporeal membrane oxygenation, or venous or arterial thrombosis at the 90-day follow-up (21). Moreover, in patients hospitalized with COVID-19 and increased D-dimer levels, in-hospital therapeutic AC with rivaroxaban or enoxaparin followed by rivaroxaban on day 30 has been recently reported to have no impact on mortality, while increasing the risk of bleeding (22).

Even though AC has been shown to prevent macrothrombotic complications (23, 24), intermediate doses did not worsen or modify the mortality outcome in these studies likely because treatment was introduced too late into the course of the disease. Indeed, it is clear that microthrombosis/lung obstruction is pathognomonic of COVID-19 worsening and hypoxemia, in contrast to most other respiratory viral infections. We previously demonstrated that pr-hospital AC (vitamin K antagonist or direct oral anticoagulant) reduces endothelial lesions (25) and also prevents worsening of COVID-19 and in-hospital mortality (15). AC regimens are probably efficient in the early stages of the disease by preventing COVID-19–associated coagulopathy and endotheliopathy. Once microthrombosis and lung obstruction have occurred, COVID-19 inexorably worsens and the AC therapy is likely to lose its protective effect on outcomes. Early AC prior to hospitalization for COVID-19 could also directly block SARS-CoV-2 entry inside targeted cells. Indeed, transmembrane protease serine 2 (TMPRSS2) and also factor Xa and thrombin can directly cleave the SARS-CoV-2 spike, enhancing viral entry (26, 27). Thus, the AC strategy needs to be proposed as a treatment option very early and before hospitalization, at the time of being a contact case or of diagnosis by an RT-PCR test. This hypothesis needs to be tested in dedicated prospective clinical studies or in preclinical models of infection.

We acknowledge some limitations in the present study. Despite efforts to control confounders by using different analytical strategies such as propensity score matching, some potential biases may have been disregarded such as the number of major bleeding events and the timing of AC therapy initiation after admission. All efforts were made to adjust the analyses for relevant variables, including cardio-vascular comorbidities, patient characteristics, and severity clinical features. We acknowledge that patients who received intermediate prophylactic dose had significantly more extensive lung damages, rates of endotracheal intubation, rates of high-flow nasal cannula use, and higher levels of CRP when compared with patients in the standard prophylactic group. Thus, it seems that intermediate prophylactic dose was started in more severe COVID-19 patients in medical wards. Moreover, in these patients, the higher prophylactic regimen of AC did not improve the outcomes.

All in all, our results highlight the futility of intermediate-dose prophylactic AC, for modification of in-hospital mortality, compared with standard-dose prophylactic AC, in patients admitted to medical wards. Our results confirm data recently published in critical patients with COVID-19. In the future, AC and its different regimens may be tested in ambulatory patients in a multicenter, randomized, controlled, open-label trial, stratified on the timing of the disease more than disease severity in already hospitalized patients.

## Data Availability Statement

The raw data supporting the conclusions of this article will be made available by the authors, without undue reservation.

## Ethics Statement

The studies involving human participants were reviewed and approved by French Data Protection Committee. Written informed consent for participation was not required for this study in accordance with the national legislation and the institutional requirements.

## Author Contributions

DS and RC designed the present study and wrote the manuscript. RC performed statistical analyses. AC and GB designed the trial. All authors reviewed the paper and have substantially contributed to the paper.

## Conflict of Interest

DS, NG, OS, AC, and RC acknowledge the following without any relation with the current manuscript. DS received consultant, lecture fees, or travel awards from Aspen, Bayer, Carmat, Alliance BMS-Pfizer, LEO-Pharma, and Boehringer-Ingelheim. NG discloses consulting fees by Boehringer-Ingelheim, Bayer, Bristol-Myers Squibb/Pfizer, and LEO-Pharma. OS received grants, personal fees, or nonfinancial support from Bayer, Alliance BMS-Pfizer, Sanofi Aventis, Daiichi Sankyo, MSD, Boston Scientifics, and Chiesi. AC received a research grant from RESICARD (research nurses) and consultant and lecture fees from Amgen, AstraZeneca, Bayer Pharma, Alliance BMS-Pfizer, Novartis, and Sanofi-Aventis. RC received Consultant fees from Aspen. The remaining authors declare that the research was conducted in the absence of any commercial or financial relationships that could be construed as a potential conflict of interest.

## Publisher's Note

All claims expressed in this article are solely those of the authors and do not necessarily represent those of their affiliated organizations, or those of the publisher, the editors and the reviewers. Any product that may be evaluated in this article, or claim that may be made by its manufacturer, is not guaranteed or endorsed by the publisher.
